# Chondrogenic Differentiation of Human Umbilical Cord Blood-Derived Unrestricted Somatic Stem Cells on A 3D Beta-Tricalcium Phosphate-Alginate-Gelatin Scaffold

**Published:** 2014-02-03

**Authors:** Masoud Soleimani, Layasadat Khorsandi, Amir Atashi, Fereshteh Nejaddehbashi

**Affiliations:** 1 Department of Hematology, Faculty of Medical Sciences, Tarbiat Modares University, Tehran, Iran; 2 Cellular and Molecular Research Centre, Ahvaz Jundishapur University of Medical Sciences, Ahvaz, Iran; 3 Department of Cell Biology, Khatam University, Tehran, Iran

**Keywords:** Mesenchymal Stem Cells, Scaffold, Chondrogenesis

## Abstract

**Objective:**

Finding cell sources for cartilage tissue engineering is a critical procedure. The purpose of the present experimental study was to test the in vitro efficacy of the beta-tricalcium phosphate-alginate-gelatin (BTAG) scaffold to induce chondrogenic differentiation of human umbilical cord blood-derived unrestricted somatic stem cells (USSCs).

**Materials and Methods:**

In this experimental study, USSCs were encapsulated in BTAG scaffold and cultured for 3 weeks in chondrogenic medium as chondrogenic group and in Dulbecco’s Modified Eagle’s Medium (DMEM) as control group. Chon- drogenic differentiation was evaluated by histology, immunofluorescence and RNA analyses for the expression of cartilage extracellular matrix components. The obtain data were analyzed using SPSS version 15.

**Results:**

Histological and immunohistochemical staining revealed that collagen II was markedly expressed in the extracellular matrix of the seeded cells on scaffold in presence of chondrogenic media after 21 days. Reverse transcription-polymerase chain reaction (RT-PCR) showed a significant increase in expression levels of genes encoded the carti- lage-specific markers, aggrecan, type I and II collagen, and bone morphogenetic protein (BMP)-6 in chondrogenic group.

**Conclusion:**

This study demonstrates that BTAG can be considered as a suitable scaffold for encapsulation and chondrogenesis of USSCs.

## Introduction

"Cartilage is an avascular tissue with low cellularity and a limited capacity for self-repair" (1). When cartilage is injured or undergone degeneration, there is little cellular invasion into the damaged region and the intrinsic repair response is insufficient to completely restore the tissue to its former functional state (1, 2). Cell transplants with and without scaffolds are used to create functional cartilage replacements in tissue engineering researches (3, 4). The adult chondrocyte has limited capacity, and this is a major challenge in providing adequate cell numbers for repair or replacement of damaged cartilage. "The *ex vivo* expansion of chondrocytes results in a loss of their phenotype" (5). Several studies have been focused on the research of biocompatible scaffolds which provide suitable three-dimensional structure and are able to support cell viability, proliferation and differentiation process (6). The appropriate choice of both cells and biomaterials represents one of the most important aspects of cell-based cartilage engineering (7, 8).

It has been reported that human umbilical cord blood stem cells can differentiated into three germ line layers (9). Recently, unrestricted somatic stem cells (USSCs) derived from umbilical cord blood are under investigation for a number of therapeutic applications (10). A number of studies demonstrate the therapeutic potential of USSCs in bone healing, reducing graft-versus-host disease, repair of myocardial infarcts and as vehicles for gene therapy (11- 16).

In comparison to haematopoietic stem cells, USSCs are rare in cord blood, but they can rapidly expand (17). Recently, three-dimensional scaffolds for cell delivery and therapy have become a major research focus in the fields of tissue engineering (18-21).

Poly (L-lactide)/poly(ε –caprolactone are the two suitable types of biopolymers for cartilage tissue engineering (22-25). However, they can induce inflammation reactions, their degradation rates usually fail to match the rate of new tissue regeneration (26, 27). Ideal properties of a scaffold for cartilage regeneration are biocompatibility, less inflammatory, and controlled biodegradability with non-toxic degradative products (28).

Recently, a porous denatured collagen scaffold, gelatin, has been used as a scaffold for cartilage tissue engineering (29, 30). The biological origin of collagen-derived gelatin makes this material an attractive choice for tissue engineering (31). It is believed that alginate and agarose lack native ligands that allows interaction with mammalian cell (32). However, these hydrogels induce minimally invasive injection of hydrogel/cell constructs for tissue engineering (33-35). We used a three-dimensional alginate/gelatin/beta-tricalcium phosphate scaffold on which the cells were able to seed without cell loss, and lay in a uniform array in palisades. In the present study, we investigated whether USSCs encapsulated in the beta-tricalcium phosphate-alginate-gelatin (BTAG) scaffold could produce cartilage tissue.

## Materials and Methods

### Generation and expansion of unrestricted somatic stem cells

In this experimental study, USSCs were generated from 30 cord blood. Both cord blood and placenta were collected from the Taleghani Hospital, Tehran, Iran, after obtaining an informed consent from donors and a protocol approved by The Ethics Committee of Department of Hematology, Faculty of Medical Sciences, Tarbiat Modares University, Tehran, Iran. The mononuclear cell fraction was obtained using Ficoll (Sigma, USA) density gradient separation, followed by ammonium chloride lysis of red blood cells. Cells were then plated out at 5-7×106 cells/ml in T25 culture flasks. Low glucose Dulbecco’s Modified Eagle’s Medium (DMEM, Sigma, USA) in addition to 30% fetal bovine serum (FBS), dexamethasone (10-7 M, Sigma, USA), penicillin (100 U/ml, Sigma, USA), streptomycin (0.1 mg/ml, Sigma, USA), and L-glutamine (2 mM, Sigma, USA) were used as media to initiate growth of the adherent USSC colonies. Expansion of the cells was also performed in low glucose DMEM with FBS. Cells were incubated at 37˚C in a humidified 5% CO2 atmosphere (36). When cells reached 80% confluency, they were detached by 0.25% trypsin/EDTA (Sigma, USA) and passaged for 3 times.

### Flow cytometry analysis

Expression of cell surface markers on the USSCs culture prior to use of chondrogenic media were analyzed using flow cytometry. The cells were characterized with regard to a set of markers characteristic for USSCs including CD29 (K20), CD49 (L25.3), CD166 (3A6) and FLK-1 (KDR) from (Abcam, USA).

### Preparation of the scaffold

BTAG scaffold was fabricated as previously described (37). Briefly, 1.6 g of alginate powder (Sigma, USA) was first dissolved in 100 ml of distilled water using magnetic stirring. Then, 1.6 g of gelatin powder (Sigma, USA) was added to the alginate solution and mixed vigorously at 50˚C to yield a homogenous solution. At the same time, 0.8 g of b-tricalcium phosphate powder was suspended in 96 ml of distilled water. Finally, with addition of b-tricalcium phosphate (Sigma, USA) suspended in distilled water to the alginate/gelatin solution, a three-component composite was produced. It should be mentioned that the final concentration of the solution was 2 weight percent (wt%), in which the proportional weight of alginate and gelatin was equal, while the proportional weight of b-tricalcium phosphate was 25% of sum of total weights of utilized polymer. Air bubbles were eliminated from the solution by addition of 2% glutaraldehyde and 0.05 M calcium chloride solution whose proportional weight was 0.25% of the alginate weight. To avoid possible cytotoxicity, a minimum amount of glutaraldehyde whose proportional weight was 0.25% of the proportional weight of gelatin was also used. The solution was frozen at -40˚C for 20 minutes and freeze-dried for 12 hours by taking the following steps: i. the homogeneous mixtures were put into 24-well polystyrene culture dishes, ii. they were rapidly transferred into a freezer at the preset temperature (20˚C) to freeze water and to induce solid-liquid phase separation, iii. the solidified mixtures were maintained at that temperature overnight, and iv. in the final stage, frozen mixtures were lyophilized (Lyophilizer, Epsilon 1-12D Christ, Germany) at 0.02 mbar and freeze-drying temperature of -40˚C for 20 minutes.

The scaffolds were characterized by a scanning electron microscopy (SEM, Tescan, USA) to determine the average pore size of the unseeding scaffold. The sizes of 20 different pores on each sample were calculated using SEM, and the mean value was reported as an approximate mean pore size.

### 3D culture system and chondrogenesis differentiation

Prior to culture initiation, the scaffolds were sterilized by 70% ethanol for 10 minutes followed by washing twice with phosphate buffered saline (PBS) (38). Five hundred thousand USSCs at passage-three were suspended homogenously in 500 μl of DMEM +3% FBS placed on the top surfaces of the scaffold cubes. Cell/scaffold constructs were placed in the wells of a 12-well culture plate under the laminar hood for 1 hour during which the drop disappeared owing to its penetration into scaffold pores. USSCs were encapsulated in BTAG scaffold and cultured for 3 weeks in chondrogenic medium as chondrogenic group and in DMEM as control group. Afterward, chondrogenic medium containing 50 ng/ml ascorbic acid 2-phosphate (Sigma, USA), 10 μM dexamethasone (Sigma, USA), 10 ng/ml transforming growth factor-beta (TGF-β, Sigma, USA), 10 ng/ml basic fibroblast growth factor (bFGF; Sigma, USA), 0.1% Insulin-Transferrin-Selenium (ITS, Sigma, USA) and1 mg/ml linoleic acid (Sigma, USA) was used and incubated at 37˚C in a humidified 5% CO2 atmosphere for 21 days. First medium replacement was done on day 3, and the subsequent medium changes were performed every 2 days.

### MTT Assay

The cell viability was measured by 3-(4, 5-dimethyl) thiazol-2-yl-2, 5-dimethyl tetrazolium bromide (MTT) assay on days 0, 1, 3, 7, 14 and 21 of cell culture in both chondrogenic and control groups. Briefly, MTT solution (5 mg/mL) was added into each 50-μl culture tube. The cells were continually cultured for another 5 hours. During this period, viable cells could reduce the MTT to formazan pigment, which was dissolved by 300 μl isopropranol after removal of the culture medium. The absorbance at 570 nm was recorded by a micro plate reader (Bio-Rad 380, USA).

### Scanning electron microscopy

Both unseeded and seeded scaffolds on days 3, 7, 14 and 21 were fixed with 2.5% glutaraldehyde buffered in 0.15 mol/L sodium cacodylate at 20˚C for one hour (pH=7.2). After fixation, the cultures were repeatedly rinsed in cacodylate buffer. The cultures were then dehydrated in a graded series of ethanol (50, 70, 95 and 100% alcohol) prior to critical point drying. The preparations were sputter-coated with gold-palladium before SEM.

### Histological and immunofluorescence

Constructs were washed in PBS, fixed with 3.7% formaldehyde in PBS overnight, dehydrated through a graded series of ethanol, embedded in paraffin, and sectioned at a thickness of 5 μm. For histological analysis, sections were deparaffinized, rehydrated, and stained with Alcian blue. For an immunofluorescence stain, rehydrated sections were pre-treated with triton-X100 for 15 minutes, incubated with rabbit polyclonal antibodies (ab34712, Abcam, UK) against types II collagen at 1:100 dilutions for 1 hour, rinsed with PBS three times for 5 minutes each, and then incubated with An Alexa Fluor®594-conjugated goat anti-rabbit IgG polyclonal secondary antibody (Abcam, UK) at 1:100 dilutions for 1 hour. After rinsing three times with PBS, sections were mounted using mounting medium containing 4’-6-diamidino- 2-phenylindole (DAPI). The staining was visualized under wavelength of 594 nm using an inverted microscope (Olympus, Japan) equipped with a digital camera (Olympus, Japan). Unseeded BTAG scaffold was used as negative control group.

### RNA preparation and reverse transcription polymerase chain reaction

Using an RNeasy Plus Mini Kit (Qiagen, USA), RNA was isolated from BTAG scaffold homogenized by Qiashredder (Qiagen, USA) according to manufacturer’s instructions. RT-PCR was performed using a One-Step RT-PCR Kit (Qiagen, USA) containing both reverse transcriptase to synthesize cDNA from the RNA isolated and DNA polymerase for the PCR. RT-PCR conditions consisted of a 30-minute step at 50˚C to allow the reverse transcriptase activity, followed by 15 minute at 95˚C to deactivate the reverse transcriptase and to activate the Taq polymerase which was present in the enzyme mixture. The PCR process consisted of a 6-second step at 94˚C (denaturing), a 30-second step at 55˚C (annealing), and a 45-second step at 72˚C (extension), while all steps were repeated for 30 cycles. Primer sequences were as follows with the expected product length: aggrecan, sense 5' GAA TCT AGC BTAG GAG ACG TC 3', antisense 5' CTG CAG CAG TTG ATT CTG AT 3' (540 bp); collagen I, sense TCC GAC CTC TCT CCT CTG AA 3', antisense 5' GAG TGG GGT TAT GGA GGG AT 3' (388 bp); collagen II, sense 5' ACC AAA GGG ACA GAA AG 3', antisense 5' ACA GCA TAA CAT GGG GCT TC 3', (470 bp); BMP-6, sense 5' CTC GGG GTT CAT AAG GTG AA 3', antisense 5' ACA GCA TAA CAT GGG GCT TC 3' (412 bp); and B2M, sense 5' TCT GGG TTT CAT CCA TCC 3', anitsense TAC CTG TGG AGC AAC CTG 3' (432 bp), which was used as a house-keeping gene.

### Statistical analysis

The data were analyzed using SPSS (SPSS Inc., Chicago, USA) version 15. A two-tailed Student’s t test was used for comparing the obtained values in either 3D culture. All measurement tests were performed at least 10 times. All values were stated as means ± standard deviations. P<0.05 was considered to be statistically significant.

## Results

### Unrestricted somatic stem cells

**Fig 1:**
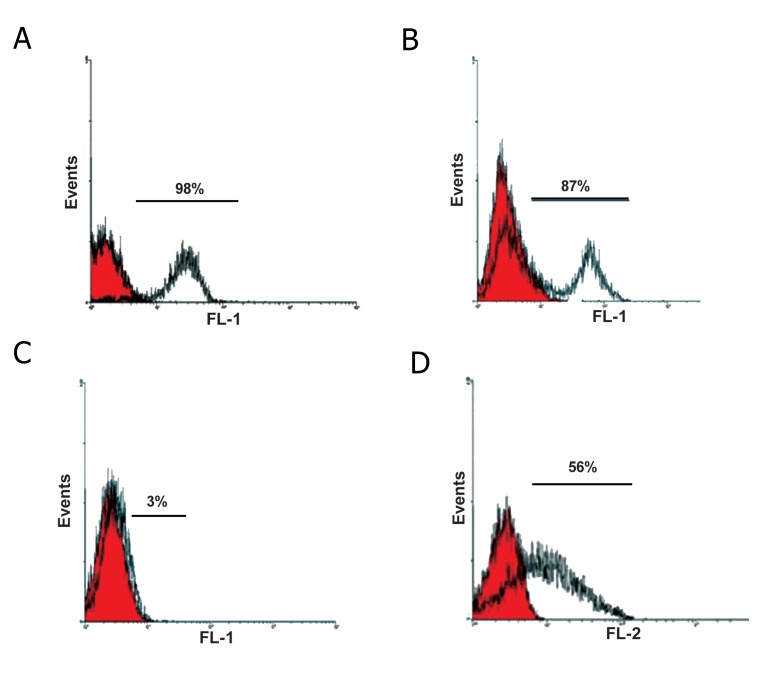
Characterization of the different surface markers. A. CD29, B. CD166, C. FLK-1 and D. CD49. Red and white histograms showed control and cell surface markers, respectively. The bars on the peak levels of histograms are a measure of positive expression of surface markers.

Cell surface markers detect by flow cytometry revealed that USSCs caused highly expression of CD29 and CD166, moderate expression of CD49, and low expression of FLK-1. The results are shown in figure 1.

### Scaffold

**Fig 2:**
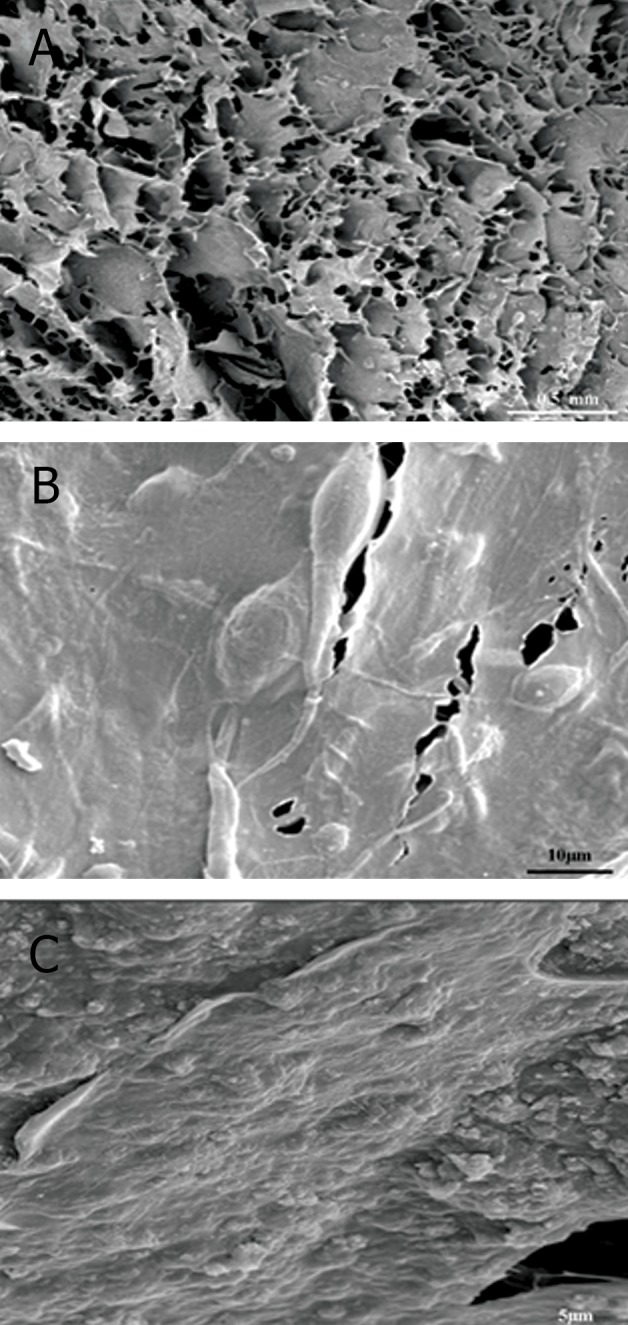
Scanning electron microscopy images of both unseeded and seeded scaffolds. A. Unseeded scaffold, B. Control group and C. Chondrogenic group.

SEM studies indicated that the unseeded spongy scaffold possessed numerous interconnected pores. The scaffolds were characterized to have the porosity and mean pore size of 318.4 μm. SEM evaluations showed that USSCs formed continuous sheets of cells, filled the interconnected pores of the BTAG scaffolds by the end of 3 weeks of cultivation both in chondrogenic and control groups. In control group, the morphologies of USSCs on BTAG were flat and elongated, while the cells in chondrogenic media showed round-shaped chondrocyte-like morphology (Fig 2).

### MTT analysis

**Fig 3:**
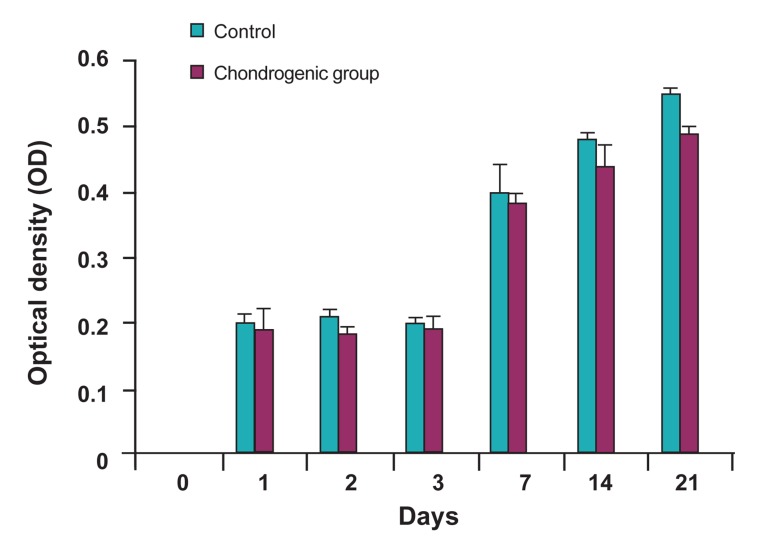
MTT results showing the metabolic activities of seeded cells on scaffold in chondrogenic medium as chondrogenic group and in DMEM as control group. P>0.05.

The cells in scaffold under chondrogenic media showed less proliferation, but there was no significant difference as compared to control group (p>0.05). The metabolic activity of USSCs within the scaffolds in each culture remained constant from days 1 to 3, indicating that no cell proliferation occurred. Between days 3 and 21, the cells in scaffolds under both culture conditions started to multiply. The results of MTT assay are shown in figure 3.

### Histological and immunofluorescence analysis

**Fig 4:**
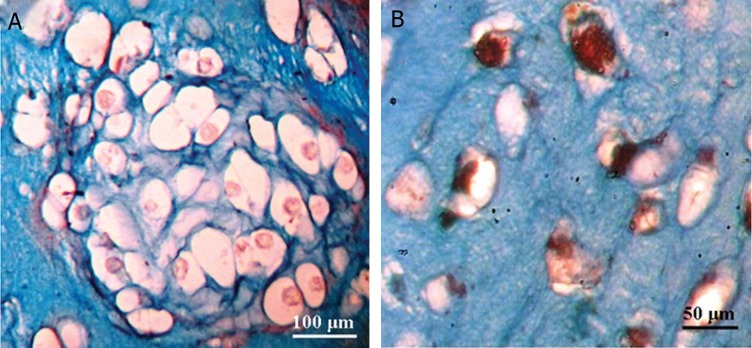
Alcian blue staining of USSCs cultured on control and BTAG scaffold.

Glycosaminoglycans (GAGs) staining by Alcian blue was intense in the chondrogenic group (Fig 4), but was not detected in the control group. In the chondrogenic group, differentiated cells in lacunae were observed during the period of 21 days.

**Fig 5:**
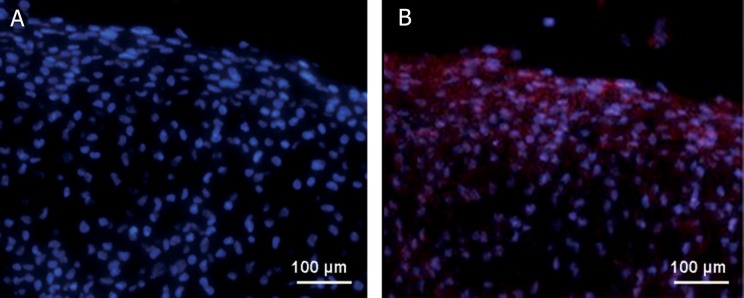
Immunofluorescence staining of collagen II (Red) with nuclear counterstain (Blue) of USSCs on BTAG scaffold. A. control group, only nucleous was stained . B. chondrogenic group, extra cellular matrix and nucleous were stained.

Collagen type II protein was not detectable by immunofluorescence method in the control group, but was strongly detected in the presence of chondrogenic media. Collagen type II protein was mainly distributed in the extracellular matrix (ECM). Cell-seeded scaffolds exhibited strong immunostaining as compared to the unseeded BTAG scaffold. On day 21, immunohistochemical staining of BTAG scaffolds for type II collagens is shown in figure 5.

### Reverse transcription polymerase chain reaction

**Fig 6:**
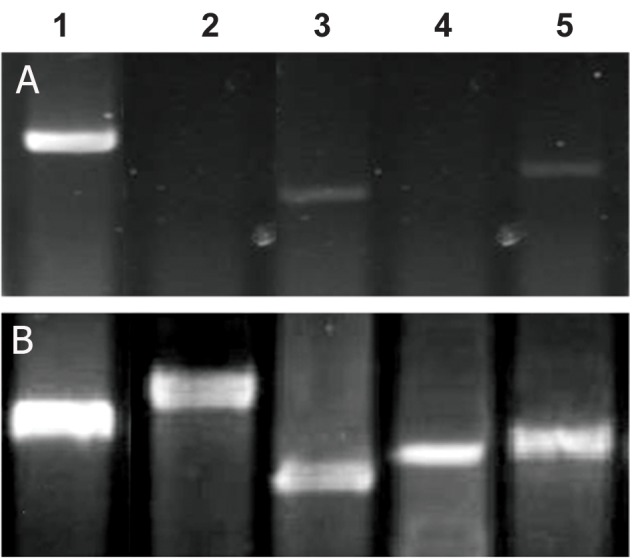
The expression of genes encoded the cartilage-specific markers in control (A) and chondrogenic (B) groups. 1; B2M, 2; aggrecan, 3; Collegen I, 4; Collagen II, 5; BMP-6.

In the chondrogenic group, mRNA expression patterns were characteristic of chondrogenesis, as demonstrated by RT-PCR. The expression levels of genes encoding bone-related proteins like collagen type I and bone morphogenic protein (BMP)-6 were greatly increased in chondrogenic group as compared to the control group. Also, the expression levels of genes encoding two other bone-related proteins including collagen type II and aggrecan were specifically induced, and sequentially increased in chondrogenic group. The results of RT-PCR are reported in figure 6.

## Discussion

In this study, we have used BTAG as a scaffold for in vitro chondrogenic differentiation of USSCs. The cell encapsulated the expressed cartilage-characteristic of extracellular matrix genes encoded the cartilage-specific markers, aggrecan, type I and II collagen, and BMP-6. The Alcian blue staining results also confirmed differentiation of USSCs into chondrocyte-like cells and secretion of GAGs in their ECM.

In previous studies synthesis of ECM components in mesenchymal stem cells in 3D culture due to round shape morphology that represent these cells differentiated into cartilage (34-36). The morphology of cells alone could not be critical in detecting chodrocyte differentiation because round chondrocyte can express type I collagen and expanded chondrocyte can express collagen type II (36, 39).

In this study, we demonstrated that the roundshaped chondrocyte-like cells differentiated from USSCs can secret GAGs and type I and II collagen in their ECM. BMP-6, known as an inducing factor for chondrogenesis, was expressed in chondrogenic group (23). Expression of BMP-6, revealed in this study, indicates that the BTAG scaffold has positive effect on this gene.

There was no significant difference in proliferation of seeded cells between chondrogenic media and control group by MTT assay, indicating that the BTAG scaffold is not toxic for USSCs.

It is well known that spherical cell morphology of mesenchymal stem cell (MSCs) relates to the synthesis of cartilage ECM components in 3D cultures. Additionally, rounded chondrocytes can synthesize type I collagen and spread chondrocytes can express type II collagen (34-39). Thus, cell shape may not be critical in influencing chondrocyte differentiation.

Seda Tigli et al. (23) isolated human chondrocytes, human embryonic stem cells (ESCs) and MSCs derived from the following three sources, human embryonic stem cells, bone marrow and adipose tissue, while being assessed for chondrogenic potential in 3D culture. They suggested that the human ESCs were the preferred cell source.

ESCs possess ethical issues limiting their application in cartilage regeneration. In addition, some reports have been shown that transplantation of ESCs can induce teratoma in the animal model (40, 41).

Chondrocytes, adipose stem cells and bone marrow mesenchymal stem cells (BMSCs) have been shown to be suitable cell sources for cartilage tissue engineering with biomaterial scaffolds such as silk and chitosan under appropriate culture conditions. Chondrocytes, BMSCs and adipose stem cells were cultured in chitosan scaffolds (42-45).

The chondrocytes lose their phenotype during expansion. It has been restricted the use of these cells for cartilage tissue engineering (46). Kogler et al. (9) have isolated USSCs, a multipotent stem cell population, from human umbilical cord blood. The USSCs have been suggested as a more immature cell type than BMSCs, by the potential to differentiate into osteoblasts, chondrocytes, adipocytes, and neurons.

Also, USSCs exhibit an extended life span and longer telomeres when compared with the BMSCs. Additionally, USSCs can be expanded up to 1015 cells without losing pluripotency in culture (47). In a study by Airey et al. (48) USSCs did not induce macroscopic or microscopic tumors 6 months after transplantation into a fetal sheep model.

The alginate hydrogels for use as a scaffold have been investigated in various researches. Although these researches revealed maintenance of the chondrocytic phenotype in vitro, but their poor biomechanical properties and handling characteristics limited their application in tissue engeenering (49-54). Recently, the ability of gelatin, a porous denatured collagen scaffold, to act as a biomaterial scaffold for cartilage tissue engineering has also been evaluated (27, 28).

Eslaminejad et al. demonstrated osteogenic differentiation of BMSCs in BTAG scaffold (37). In present study, chondrogenic effects of this type of scaffold were shown. Combination of gelatin and alginate as BTAG scaffold may show better result than gelatin- or alginatealone for cartilage tissue engineering. To clarify this, we suggest a comparative study of BTAG, gelatin and alginate scaffolds in chondrogenic differentiation of various MSCs.

## Conclusion

The results of this study demonstrated that BTAG scaffold provides a suitable environment for differentiation of USSCs to chondroblasts and cartilage tissue regeneration. Extrapolation of these data to the human situation is not appropriate. However, this information does provide a stimulus for true clinical investigations.
